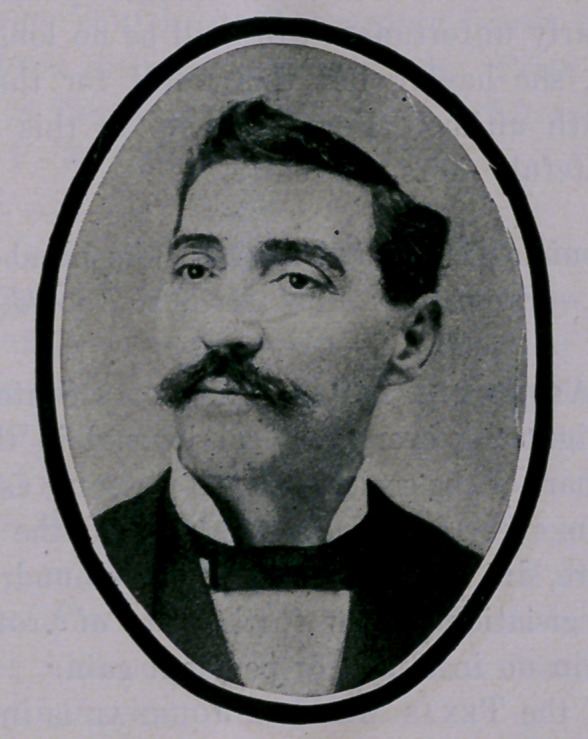# News and Miscellany

**Published:** 1905-08

**Authors:** 


### News and Miscellany.

YELLOW FEVER.—I have nothing to say on this subject at present, inasmuch as any details long since would have ceased to be "news" before this is in print. The daily papers give the news. I will only say that since the fact has been established that the mosquito is the only known method of spreading the fever, more strenuous quarantine measures are resorted to than before. Instead of shotguns, it is now the United States army and navy, and State troops; trade and travel are stopped, even United States mail, or else fumigated (!!) and at Shreveport a stranger who had "slipped into town" was whipped!!!! and still the fever has spread to every Southern State except Texas.

DR. G. FRANK LYDSTON, of Chicago, has been elected a fellow of the London Society of Authors, an honor which has come to very few American writers. This is a graceful recognition on the part of the Londoners of a very brilliant author who, in addition to being remarkably versatile, is intensely interesting.—*Buffalo Medical Journal.*

Lydston should be one of the Trustees of the American Medical Association. I nominate him now.

DISINFECTING THE CONFESSIONAL.—On the advice of the Mexican Board of Health, the government has issued an order for the daily disinfection of confessionals in all the churches of the capital. Priests neglecting the order are subject to fine and imprisonment. According to the health board, the confessional may be an active means of spreading contagious and infectious diseases, and its purification is important in controlling epidemics.—*New York Medical Record.*

The Germicide Gas Generator (formaldehyde) is the thing to do it.

"THIS HERE JONES" AGAIN.—The *New York Medical Journal* has administered a merited rebuke to the *California State Journal of Medicine* for a libel which the latter published concerning the former. The editor of the California *Journal* has made an abject apology for his discourteous article and we presume this will end the matter.

In its issue of July 1, the *New York Medical Journal* publishes the correspondence in full, together with editorial comments, under the heading "A Warning to Calumniators." The action of our esteemed contemporary merits and should receive the approbation of every medical journal in the country. Some have already commented upon the incident and others no doubt will do so. The St. Louis *Medical Review,* July 8, in an extensive commentary, says in conclusion: "The *California State Journal of Medicine* is a little given, as a continuous performance, to the search of motes in the eyes of others." Perhaps the sophomoric editor ("This here Jones") will now close up his "continuous performance" show in view of the castigation he has received.—*Buffalo Medical Journal.*

---

PROF. MARVIN L. GRAVES, M. D., the new Professor of Theory and Practice of Medicine, Medical Department University of Texas, Galveston, Texas, recently appointed by the Board of Regents, vice J. W. McLaughlin, M. D., resigned.

Dr. Graves was born in McLennan County, Texas; graduated M. D. Bellvue Hospial Medical College, 1891; was appointed Superintendent Southwest Texas Insane Hospital, San Antonio, in January, 1898 (Sayers), which position he filled with distinction until he was chosen to fill the Chair of Medicine in the State University Medical College.

---

DR. H. W. COE, of Portland, Oregon, editor of the *Medical Sentinel,* was elected President of the Association of American Medical Editors at the recent Portland meeting.

Dr. F. E. Daniel, of the TEXAS MEDICAL JOURNAL, better known as the "Texas Red Back," shows us what hot weather in the extreme South may do, in an editorial on "The Chicago Octopus: The Journal of the American Medical Association." The casual reader who opens the scorching covers of the "Red Back" with expectations of warm material within, is not doomed to disappointment. This particular editorial is sufficiently sulphurous to suit the most highly seasoned palate and, incidentally, it is written so frankly as to be quite convincing. A trifle too peppery for pleasant summer reading, it may serve, however, for thoughtful meditation when the cooler days come on.—*Chicago Clinic.*

---

T. O. MAXWELL, M. D., the new Superintendent Southwest Texas Insane Asylum, San Antonio, recently appointed, vice Dr. Graves, now Professor of Medicine in the Medical Department of the University of Texas.

Dr. Maxwell, for ten or twelve years, has been first assistant physician to the Texas Insane Asylum at Austin. He was born at Abingdon, Va., September, 1855. M. D. from Medical Department of the University of Virginia, 1876, and from Vanderbilt, 1878 (valedictorian).

---

"Full many a man both young and old
Has gone to his sarcophagus
By pouring water icy cold
Adown his hot esophagus."

Gratitude Expressed in Babu.—Dr. Margaret H. Norris, the physician in charge of the Sarah Seward Hospital at Allahabad, in India, furnishes beautiful evidence of the gratitude of the natives among whom she and many other American women or working in a medical way. The two letters subjoined are genuine, and from the husbands of women who had been patients of Dr. Norris:

"Dear She:—My wife has returned from your hospital cured. Provided males are allowed at your bungalow, I would like to do you the honor of presenting myself there this afternoon, but I will not try to repay you; vengeance belongeth unto God. Y'rs, noticeably,

"______ _________"

"Dear and Fair Madam:—I have much pleasure to inform you that my dearly unfortunate wife will be no longer under your kind treatment, she having left this world for the other on the night of the 27th ultimo. For your help in this matter I shall ever remain grateful. Y'rs reverently,

"______ _________"

These testimonials are printed in the India number of *Woman's Work* for April.—From *New York Medical Record.*

---

Revolt all Along the Line.—The Texas State Society made the mistake of ignoring every medical journal in the State—that is, the delegate part of the society—much the same as in Ohio. The journals have in every instance been loyal to the State Society, and there, as here, did more than any score or hundred other members to obtain legislation for the furtherance of professional honor and profit, and in no instance for personal gain.

The editor of the Texas Medical Journal is in the right and he will win. Dr. Edwards, of the *Virginia Medical Semi-Monthly,* came out of the professional struggle in Virginia victorous because he was in the right. There is a whole lot of irritation in North Carolina, to say nothing of the split in New York.

The Iowa profession has had a walk-out. The Virginia physicians never did take to the reorganized association, but held aloof from the delegate-councilman rule and have constantly stood out against the blandishments of the Association's hired man.—*Culberson's Editorial in Lancet Clinic.*

---

It Kills Them.—Prof, Allen J. Smith, late Professor of Pathology, Medical Department University of Texas, now of the University of Pennsylvania, put a slide containing a culture of tubercle bacilli in a sealed envelope in the pocket of a coat hanging up in the room; and another he wrapped in two thicknesses of billiard cloth, and turned loose an immense volume of formaldehyde on them. In a very short while it killed them, as it did also the bacilli of diphtheria and the sporulating anthrax bacilli, the toughest of all pathogenic germs. The apparatus used was the Germicide Gas Generator. The formaldehyde is generated rapidly direct from the wood alcohol through a copperized disc. No danger of fire,—a child can operate,—no keyhole "contrapsion",—no moisture to ruin wall paper. It penetrates mattresses, bedding, carpets, rags, clothing, all *in situ* and thoroughly disinfects everything in a room. Every doctor should own one. Pays for itself by renting it. Every room in which a consumptive lives, or has died, every school room, every church, ward, boarding house, courthouse, jail, and hotel should be disinfected at intervals. Price $25, by express paid anywhere in Texas on receipt of price. Literature on request. Address: Dr. F. E. Daniel, Austin, Texas.

---

Dr. Marvin L. Graves, Superintendent Southwest Texas Insane Asylum, San Antonio, Texas, has been elected by the Regents of the University of Texas to the Chair of Practice of Medicine, made vacant by the resignation of Dr. J. W. McLaughlin.

Dr. Thomas O. Maxwell, for many years first assistant physician to the (Austin) Texas Insane Asylum, has been appointed by the Governor to fill the vacancy of Superintendent of the Southwest Texas Asylum, vice Graves, resigned, and Dr. J. H. Eastland, formerly of Waco, late assistant physician to the Texas Epileptic Colony at Abilene, has been appointed first assistant physician at the Austin Asylum, vice Maxwell.

These are all excellent appointments and the Journal congratulates the State and rejoices at the deserved rcognition and promotion of these able and popular young physicians.

## Figures and Tables

**Figure f1:**
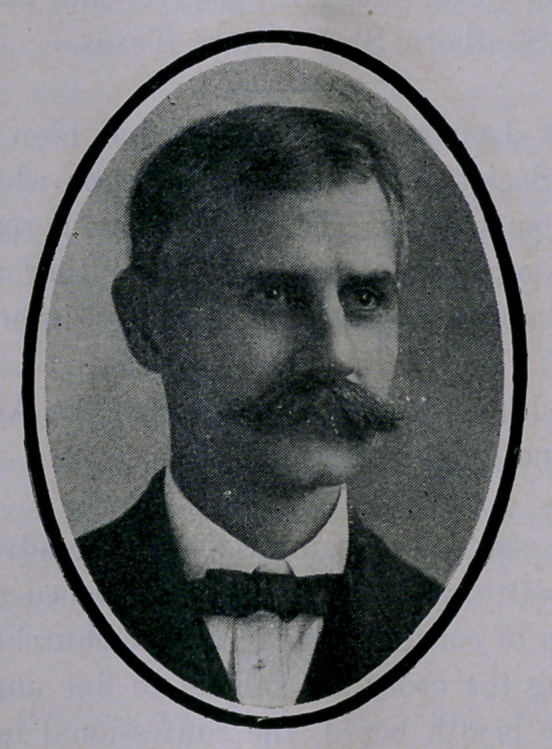


**Figure f2:**